# Analysis of the stress distribution of the subtalar joint and fusion efficacy after double-screw insertion

**DOI:** 10.1186/s13018-018-1034-4

**Published:** 2019-01-14

**Authors:** Cheng-song Yuan, Jing-jing Sun, Si-ya Wu, Guo-qing Jing, Mei-ming Xie, Kang-lai Tang

**Affiliations:** Department of Orthopaedic Surgery, Southwest Hospital, the Third Military Medical University, No. 30, Gaotanyan Street, Chongqing, 400038 China

**Keywords:** Subtalar joint, Three-dimensional finite element method, Subtalar fusion, Cannulated screw, Clinical follow-up

## Abstract

**Background:**

Screw fixation is a typical technique for the isolated subtalar joint. However, no consensus has been reached on how to select the most suitable insertion position and direction. This study aims to find the ideal screw insertion and then explore its influence on the clinical efficacy of subtalar fusion by analyzing the effects of different cannulated screw insertions on the stress distribution, anti-rotary strength, and anti-inversion/eversion strength of the subtalar joint.

**Methods:**

In this study, we investigated three cannulated screw insertions for subtalar fusion: screw insertion with the most uniform stress distribution (group A), lateral-medial parallel screw insertion (group B), and traditional longitudinally parallel screw insertion (group C). The effects of these three insertions on the loading stress of the subtalar joint (including stress distribution, anti-inversion/eversion strength, and anti-rotary strength) were comparatively analyzed with the three-dimensional finite element method to screen the ideal screw insertion. Moreover, a prospective study was conducted to analyze the influence of the ideal screw insertion on subtalar fusion, including the fusion rate, fusion time, and clinical efficacy (VAS score, AOFAS score, and complications).

**Results:**

Group B was worse than group A with respect to the stress distribution uniformity, but slightly better than group C, and better than both groups A and C in terms of the anti-rotary strength and anti-inversion/eversion strength. The screw insertion based on the most uniform stress distribution is not feasible in surgery. Therefore, the lateral-medial antiparallel screw insertion is the ideal insertion. From January 2012 to June 2016, 48 cases were treated by subtalar fusion with the ideal screw insertion, and then followed up for 30.6 months (12–48 months). The fusion was proved in all 48 cases with a fusion rate of 100% by X-ray or CT scan. The mean time of fusion was 12.8 weeks (12–16 weeks). The VAS score decreased from 6.00 before operation to 1.03 on the last visit (*P* < 0.05), and the AOFAS score increased from 57.0 to 85.6 (*P* < 0.05), with a good and excellent rate of 95.8%.

**Conclusions:**

The lateral-medial parallel screw insertion not only demonstrates a good stress distribution profile of the subtalar joint but also has advantages such as easy localization and operation during surgery, as well as a high fusion rate and few complications after surgery. Therefore, it is a safe, accurate, and effective fixation mode that is worthy of being popularized clinically.

## Background

Subtalar fusion is the gold standard therapy for severe subtalar arthritis [1] to mainly relieve pain, stabilize the subtalar joint, and correct hindfoot alignment [2]. The key surgical factors influencing the efficacy of subtalar fusion are the operation approach [3], cartilage removal [4], bone graft use [5], and fixation mode [6]. The fixation mode plays an essential role in the realization of subtalar fusion [7]. There are several fixation modes, such as Kirschner wire, external fixation, and intramedullary pin and screw [8]. With the deep understanding of the kinematics and biomechanics of the subtalar joint and the improvement of fixation, screw insertion has become the first choice of fixation in subtalar fusion [9], but there are some issues such as a low fusion rate and no unified fixation mode. Easley and colleagues reported a nonfusion rate of 33% [7], mainly caused by the incorrect selection of internal fixation and the ineffective fixation mode during fusion [10]. Therefore, the implantation direction and position, as well as the quantity of screws, play key roles in the fusion rate of the subtalar joint, and double-screw insertion is the most common fixation mode, because one screw fails to defend the rotary stress of the subtalar joint and insufficient space is available for the implantation of three screws [11]. However, there are no studies on the position and direction of double-screw insertion for subtalar fixation worldwide, and there is no established double-screw insertion mode.

Based on the above issues and with the purpose of providing a good, feasible screw insertion approach for subtalar fusion and thus obtaining a satisfactory fusion rate, this study was planned to first identify the screw insertion with the most uniform stress distribution of the subtalar joint by 3D finite element analysis. Then, this study comparatively analyzes the differences of three screw insertions in terms of the stress distribution, anti-rotary strength, and anti-inversion/eversion strength to screen the ideal screw insertion and finally further analyzes the effects of the ideal screw insertion on the efficacy of subtalar fusion in a prospective form.

## Materials and methods

### Establishment of the subtalar three-dimensional (3D) finite element model

#### Collection of 2D data

A male patient was selected who was 28 years old with a height of 160 cm and a body weight of 52 kg. X-ray examination: No improper lower limb alignment and abnormal ankle bony structure were detected by ankle X-ray posterior-anterior and lateral projection scans; foot tumor, deformation, and other lesions were excluded.

CT examination: The scan range was from 5 cm above the ankle to the planta pedis of the whole feet. The scan parameters were as follows: tube current of 100 mA, tube voltage of 120 kV, and slice thickness of 0.75 mm. The up-down plain scan was performed to obtain the 2D CT images of 527 slices. The 2D CT imaging data were processed in the PACS image processing workstation system, and all data were saved in the DICOM format.

#### Construction of continuous 2D images

The raw data in the DICOM format were read by Menu bar > File > import image in the Mimics17.0 software, and the coronal and axial continuous 2D images were constructed with the CT sagittal data.

#### Threshold segmentation

Via Menu bar > Segmentation > Thresholding, the CT threshold of the bony structures in the whole feet was automatically segmented and extracted as 226-2710HU, and the CT value of the bony structures was read.

#### 3D reconstruction and segmentation

The 3D model was calculated by Segmentation > Calculate 3D, and the talus and calcaneus entities were segmented separately by Segmentation > CT bone segmentation.

#### Establishment and assembly of the subtalar joint 3D model

The talus and calcaneus entities were duplicated and reversely translated 50 mm along the reference axis. Then, the talus and calcaneus models were migrated opposite along the reference axis, and Boolean operations were performed on the talus and calcaneus entities to delete the original talus and calcaneus entities separately and save the intersection community. Finally, the whole 3D entity models of the talus and calcaneus were generated and output in a format of “x_t” for use in the subsequent finite element analysis.

#### Screw treatment

This study uses a simplified model and hardly considers the screw threads. Therefore, the details of the screw threads were neglected, and the screws were simplified as 7.3 mm × 8 cm glossy cylinders.

### Experimental grouping and computation

#### Effects of three screw insertions on the stress distribution of the subtalar joint

Group A: The screw insertion with the most uniform stress distribution of the subtalar joint was analyzed.

The ranges for the screw insertion were set as follows: (1) two screws penetrated the subtalar joint; (2) the bony areas for the screw entry were the bone surface of the talar neck (between the anterior ankle joint line and the talonavicular joint line) and areas of posterior and posterosuperior calcaneal tubercles; and (3) the screw insertion with the most uniform stress distribution of the subtalar joint was found by changing the distance between two screws and the angle between the screw and the subtalar joint and loading a stress of 50 Mpa on the calcaneus along the screw.

Group B: The effects of lateral-medial parallel screw insertion on the stress distribution of the subtalar joint were analyzed.

Procedures of the lateral-medial parallel screw insertion: First, the talar neck and posterior calcaneal tubercle were divided in triplicate transversally. Second, two screws were inserted separately from the lateral-medial equal division points of the talar neck into the corresponding lateral-medial equal division points of the posterior calcaneal tubercle. Finally, a stress of 50 Mpa was loaded on the calcaneus along the screw, and thereafter, the stress distribution of the subtalar joint was observed.

Group C: The effects of the traditional anteroposterior longitudinally parallel screw insertion on the stress distribution of the subtalar joint were analyzed.

Procedures of the traditional anteroposterior longitudinally parallel screw insertion: First, the posterior calcaneal tubercle was divided in triplicate longitudinally. Second, two screws were inserted separately toward the talus via the subtalar joint from the equal division points of the posterior calcaneal tubercle. Finally, a stress of 50 Mpa was loaded on the calcaneus along the screw, and thereafter, the stress distribution of the subtalar joint was observed.

The stress distribution nephogram, uniform stress, and maximum uniform area were used as indicators to evaluate the stress distribution. A bigger uniform stress and maximum uniform area indicated a more uniform stress distribution.

#### Effects of three screw insertions on the anti-rotary strength and anti-inversion/eversion strength of the subtalar joint

Comparison of anti-inversion/eversion strength: The subtalar joint inversion was simulated by confining the calcaneal body and then exerting an upward constant traction force on the body laterally after two screws were inserted. The distance of the talus from the subtalar joint was compared, and a smaller distance suggested a higher anti-inversion/eversion strength.

Comparison of anti-rotary strength: Similarly, the subtalar joint rotation was imitated by exerting a horizontally constant traction force on the talar neck laterally. The distance of the talus from the lateral border of the subtalar joint (which served as the reference) was compared, and a smaller distance also reflected a higher anti-rotary strength.

The horizontal and vertical displacements between the talus and calcaneus were evaluated with an indicator of “mm.” A smaller maximum displacement corresponded to a higher anti-inversion/anti-rotary strength.

### Clinical data

#### Study objects

This was a prospective study approved by the Ethics Committee of Southwest Hospital. Before operation, X-ray, CT, and MRI scans, as well as the AOFAS scoring, VAS scoring, and conventional admission evaluation, were routinely performed.

The inclusion criteria are as follows: (1) severe subtalar arthritis, i.e., peripheral pain of the subtalar joint, aggravated during walking, seriously influencing daily life and (2) no efficacy of drug, local block, physical rehabilitation, and other conservative treatments for over 6 months. The exclusion criteria are as follows: (1) severe internal diseases, (2) severe subtalar infection, and (3) severe osteoporosis.

From January 2012 to June 2016, a total of 48 cases of severe subtalar arthritis were treated by subtalar fusion with the ideal screw insertion. Before operation, the course of the disease was 32 months (1–360 months), and the age was 43.7 years (19–74 years).

#### Surgical procedures

**Fig. 1:**
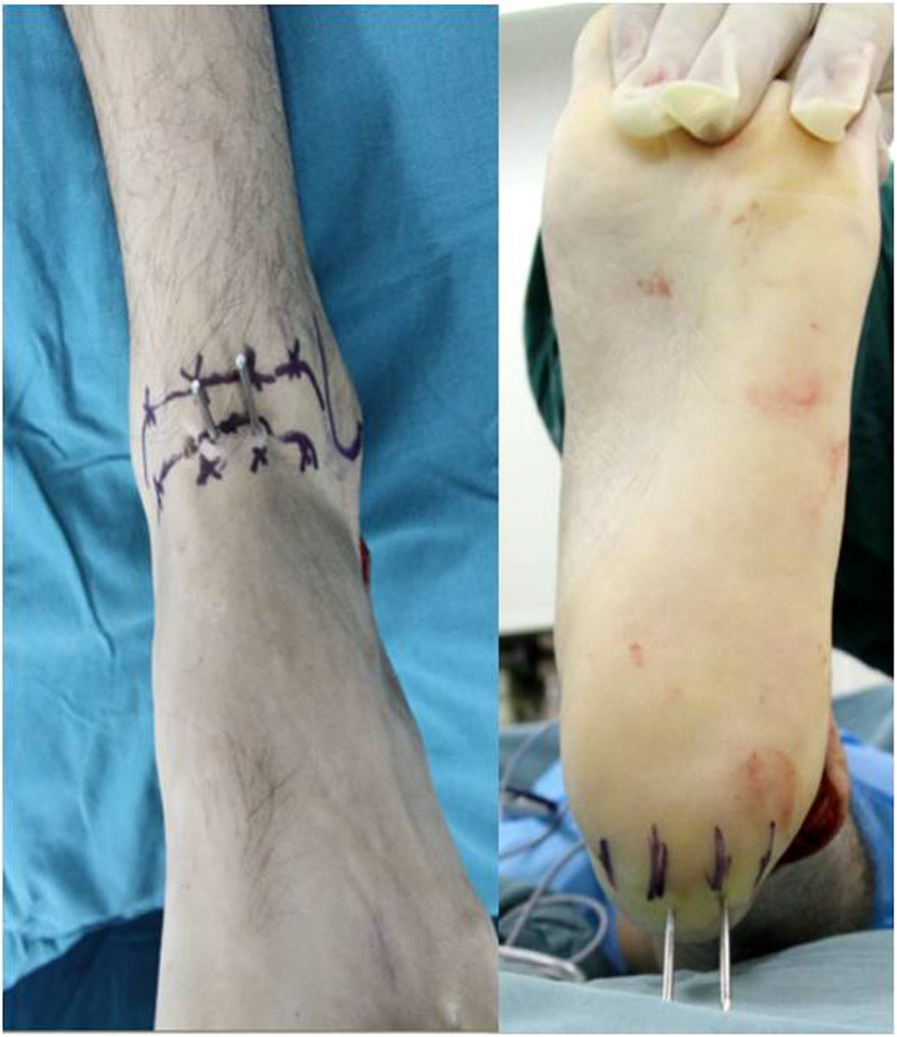
The talar neck and posterior calcaneal tubercle were divided in triplicate transversally, and two guide wires were separately inserted from the talar neck into the posterior calcaneal tubercle in equal division points

After satisfactory anesthesia, an incision was made from the lower part of the fibular apex to the basilar part of the fourth metatarsus, and the complete cartilage was removed to approximately 2 mm of the subchondral bone with osteotome and osteotribe, so that the joint surfaces were completely coupled. The subtalar joint was fixed with the ideal screw insertion (lateral-medial parallel screw insertion). The talar neck and posterior calcaneal tubercle were divided in triplicate transversally, the foot was kept in a neutral position, two 2.0 mm guide wires were inserted separately from the lateral-medial equal division points of the talar neck into the corresponding lateral-medial equal division points of the posterior calcaneal tubercle (Fig. 1), and two 7.3-mm full-thread cannulated screws were inserted in the abovementioned way. After the screws were confirmed to be in a good position by C-arm CT, the guide wires were withdrawn, the negative drainage tube was indwelled in the wound, and then the wound was sutured layer by layer.

#### Functional follow-up before and after operation

At 6 weeks, 3 months, 6 months, and on the last visit after operation, the functional evaluation and satisfaction evaluation were performed separately with the VAS pain scoring and AOFAS hindfoot function scoring. The ankle-hindfoot function scoring results were classified into four levels: level 1, 90–100, excellent; level 2, 72–89, good; level 3, 41–71, medium; and level 4, 1–40, poor. The excellent and good results were satisfactory, and the medium and poor results were unsatisfactory. At 1 day, 6 weeks, 3 months, 6 months, 1 year, and thereafter every half a year after operation, X-ray examination was performed; CT examination were performed if the fusion could not be assessed by X-ray examination. The postoperative complications were observed.

#### Statistical analysis

The measurement data were presented as $\overline{x}\pm s$. The SPSS 13.0 statistical software was used. The AOFAS score and VAS score before and after operation were analyzed by pairwise *t* test. *P* < 0.05 suggested that a difference was statistically significant.

## Results

### Establishment of the subtalar joint 3D finite element models and three different screw insertion models

The finite element models for the subtalar bony structure and double-screw fixation and three different screw insertion models were effectively established. There were 120,000 grids of information generated for the subtalus, 70,000 grids of information for the calcaneus, and 20,000 grids of information for the screws. The established subtalar joint model had a good morphology and good coupling; thus, it could be used to analyze the stress distribution of the subtalar joint.

### Three-dimensional finite element analysis data

**Fig. 2:**
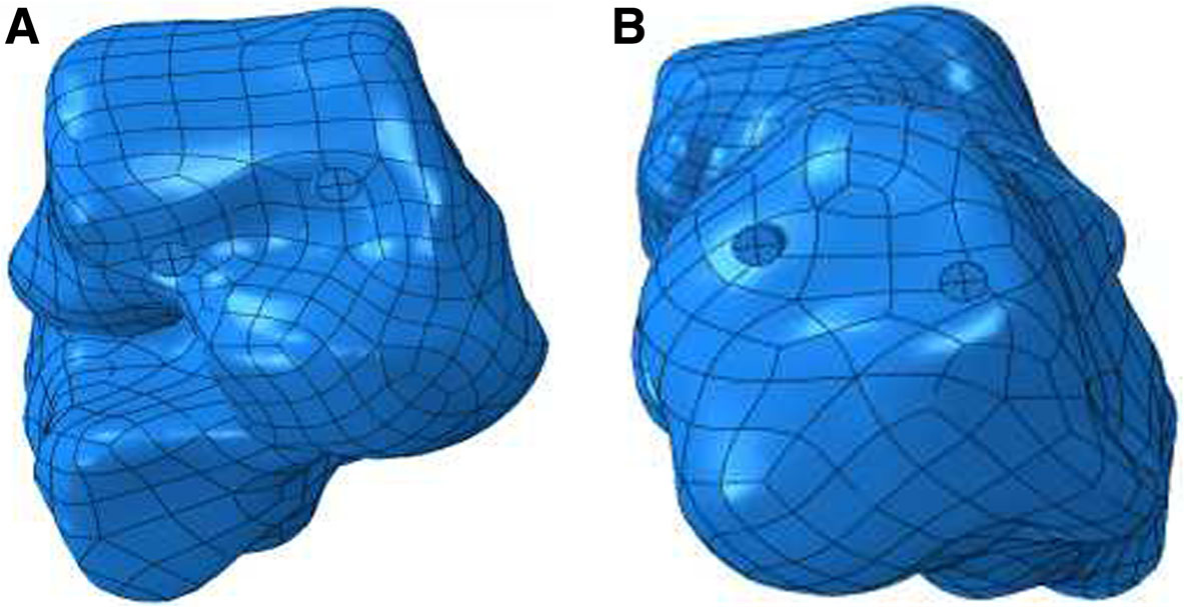
Screw insertion with the most uniform stress distribution of the subtalar joint. **a** Screw position in the subtalar neck. **b** Screw position in the posterior calcaneal tubercle

**Fig. 3:**
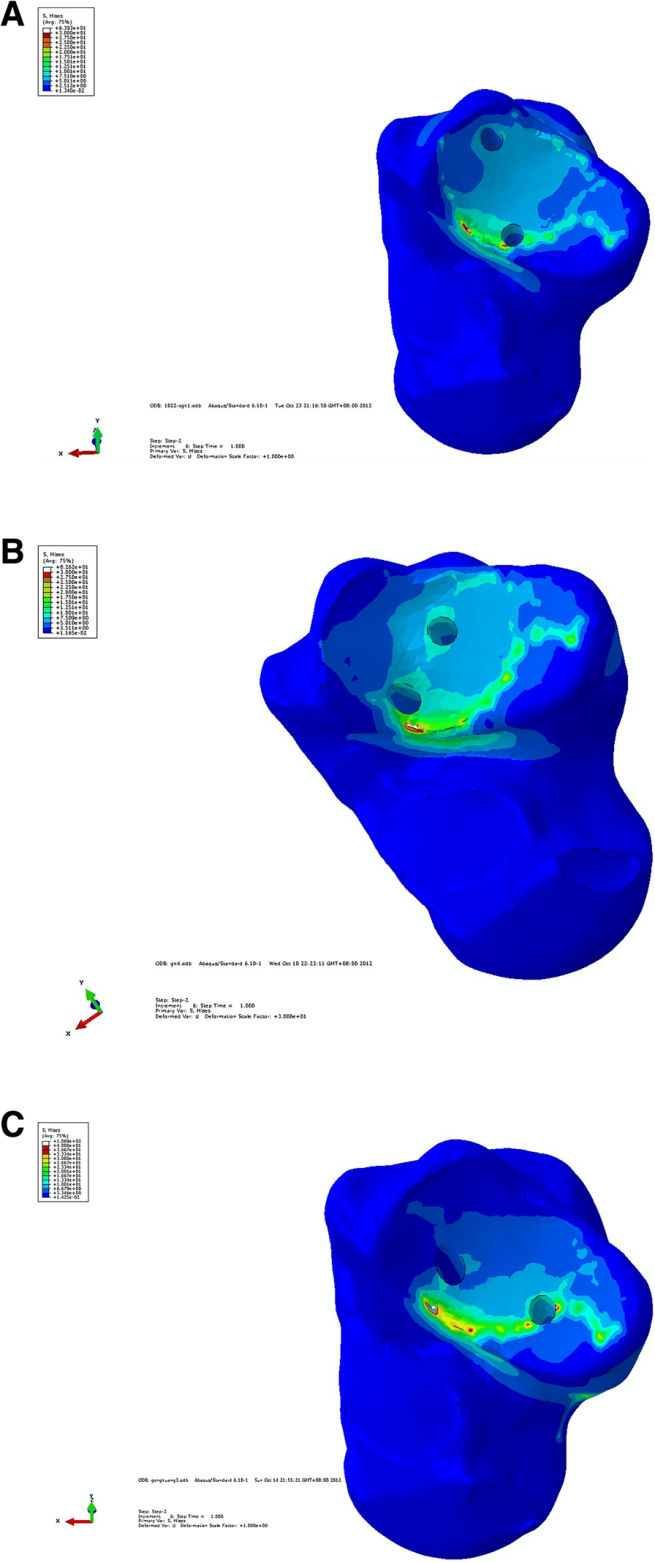
**a** Stress distribution chart of groups A. **b** Stress distribution chart of group B. **c** Stress distribution chart of group C

In group A, for the insertion in the area of the talar neck, the lateral screw was near the boundary of the talar neck, and the medial screw was close to the talar surface of the ankle joint. For the insertion in the area of the posterior calcaneal tubercle, the lateral screw was located at the boundary of the posterior calcaneal tubercle, and the medial screw was adjacent to the Achilles’ tendon terminal (Fig. 2). The distance between the two screws was 1.2 cm, the angle between the screws and the subtalar joint was 45°, the uniform stress for the subtalar joint was 7.510 MPa, the maximum uniform area was 79.56 mm [2], the maximum peak stress was 14 Mpa, and the stress ranged from 12 to 14 Mpa. In group B, the uniform stress of insertion for the subtalar joint was 7.509 MPa, and the maximum uniform area was 56.32 mm [2]. The stress was uniformly distributed surrounding the two screws but was significantly higher at the boundary of the medial screw, and it was approximately 22~25 Mpa; the maximum peak stress was 30 MPa. However, the stress concentration range was wider than the range of screw insertion with the most uniform stress distribution; thus, the stress distribution was inferior to that of the screw insertion in group A. In group C, the uniform stress of insertion for the subtalar joint was 6.679 MPa, the maximum uniform area was 51.60 mm^2^, the overall stress distribution was not uniform, the maximum peak stress was 40 MPa, the areas with a higher stress were distributed in a band shape, and the stress was 23~27 MPa; so, there was a wide range of stress concentration (Fig. 3) (Table 1).

**Table 1:** Comparison of stress distribution of subtalar joint among three screw insertion groups

	Uniform stress (MPa)	Maximum uniform stress area (mm^2^)	Maximum peak stress (Mpa)	Stress range (Mpa)
Group A	7.510	79.56	14	12–14
Group B	7.509	56.32	30	22–25
Group C	6.679	51.60	40	23–27

In groups A, B and C, subtalar joint inversion was simulated by exerting an upward constant traction force on the talar body laterally after screw insertion, and the distance of the talus from the subtalar joint was 6.061 × 10^−3^ mm, 5.798 × 10^−3^ mm, and 6.080 × 10^−3^ mm, respectively. The subtalar joint rotation was imitated by exerting a horizontally constant traction force on the talar neck laterally, and the distance of the talus from the lateral border of the subtalar joint (which served as the reference) was 7.076 × 10^−3^ mm, 6.257 × 10^−3^ mm, and 7.145 × 10^−3^ mm, respectively (Table 2).

**Table 2:** Comparison of anti-rotary strength and anti-inversion/eversion strength among three screw insertion groups

	Anti-inversion-maximum vertical displacement (× 10^−3^ mm)	Anti-rotation-maximum horizontal displacement (× 10^−3^ mm)
Group A	6.061	7.076
Group B	5.798	6.257
Group C	6.080	7.145

In group A, during the actual operations of screw insertion, inserting the lateral screw easily caused the split of the lateral talar and calcaneal bones, while inserting the medial screw easily injured the articular capsule, talar cartilage, and Achilles’ tendon terminal. It was thereby seen that the stress distribution for the subtalar joint was mostly uniform, but the surgical procedures had disadvantages such as difficult localization and insertion, easy screw exposure, a big injury to the adjacent tissues in the insertion area, and hard realization. In terms of the stress distribution uniformity, anti-rotary strength, and anti-inversion/eversion strength, the screw insertion in group C was worse than that in group B. In summary, the screw insertion in group B was the ideal insertion.

### Imaging assessments

**Fig. 4:**
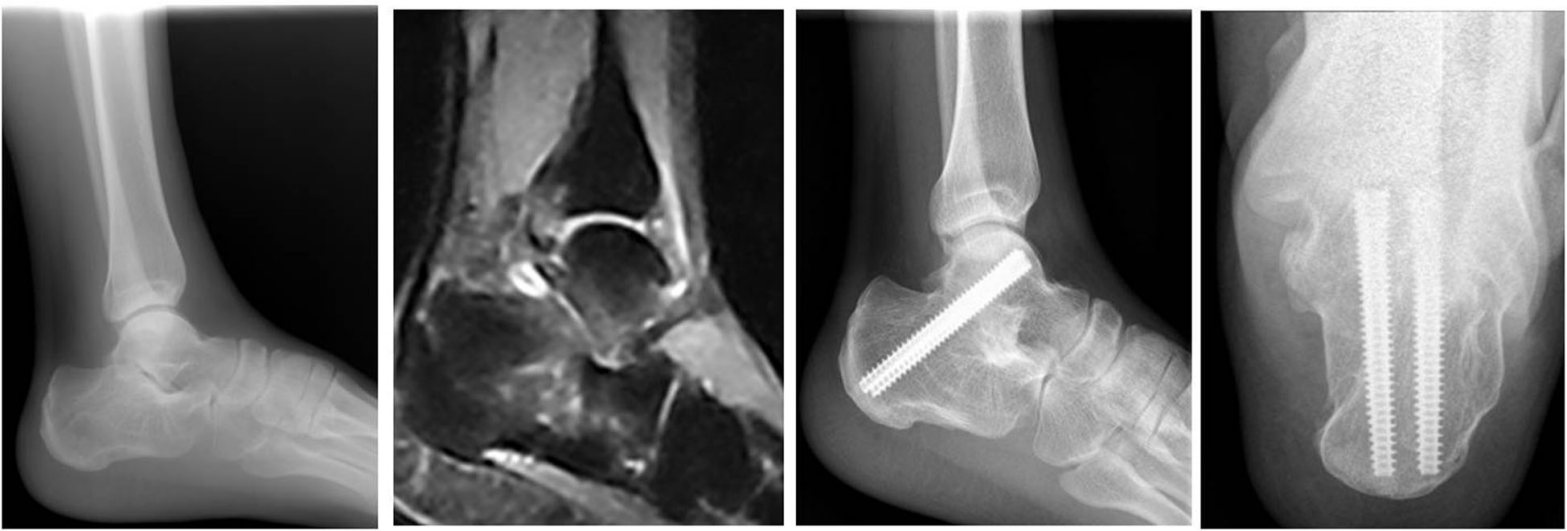
This was a typical case: at 1 year after operation, bony fusion of the subtalar joint was realized, and bone trabeculaes penetrated the subtalar joint with clear markings

Forty-eight patients were effectively followed up for 30.6 months (12–48 months); X-ray or CT scan showed bony fusion and good bony suitability of the subtalar joint in 48 cases (Fig. 4), with a fusion rate of 100% and mean fusion time of 12.8 weeks (12–16 weeks). The mean partial load time was 7.2 weeks.

### Functional outcome assessments

The mean AOFAS score was increased to 85.6 on the last visit from 57.0 before operation, while the mean VAS score was decreased to 1.03 from 6.00 (Table 3), and the differences were statistically significant. The postoperative scores were excellent in 42 cases, good in 4 cases, medium in 1 case, and poor in 1 case, with an excellent and good rate of 95.8%. Forty-five cases reported satisfactory operation results, with a satisfaction rate of 93.7%.

**Table 3:** Comparison of AOFAS score and VAS score before and after operation

	Preoperative	At the last visit	*P* value
AOFAS score	57.0 ± 4.6	85.6 ± 5.4	0.021*
VAS score	6.00 ± 1.2	1.03 ± 1.3	0.001*

*Compared with preoperative, *p* < 0.05

### Complication assessments

There was wound numbness in 1 case and hindfoot pain in 2 cases, and no complications such as skin necrosis, infection, as well as screw loosening and breakage were observed.

## Discussion

3D finite element analysis (FEA), expanded to the medical life science field from the structural engineering field, has become a practical, efficient, and digital analysis approach. FEA can be applied to effectively simulate the true geometry of the ankle and the stress distribution of various ankle parts [12]. The subtalar joint is complex in anatomy and biomechanics, and most of the actual mechanical issues are difficult to solve accurately; however, the different stress distribution profiles of various subtalar sections in a complicated shape can be resolved by the high-precision mechanical computation of 3D FEA [13]. In this study, a subtalar joint model was established for double-screw insertion. With the established model, the stress and displacement distribution profiles in different experimental groups were investigated, and the screw insertion with the most uniform stress distribution of the subtalar joint was analyzed to serve as the control group. Then, the effects of three screw insertions on the stress distribution, anti-inversion/eversion strength, and anti-rotary strength of the subtalar joint were comparatively analyzed, and the ideal, feasible screw insertion was screened by combining the difficulties during the actual operation. Penix and colleagues [14] first introduced FEA to orthopedic research. Subsequently, FEA was gradually applied in a wide range of research on orthopedic biomechanics, and it can be used to analyze the internal stress changes of bones and joints. In particular, it is uniquely advantageous in analyzing the influence of different internal fixation modes on the stress distribution of bones and joints. However, there are rarely any studies on the application of FEA in the ankle. The biomechanics of the ankle were previously studied using a pressure-sensitive membrane, pressure sensing platform, and pressure sensor, but the internal stress of the joint and bone cannot be precisely measured, because the feet have many small bones and it is difficult to fix the mechanical measurement brace. In the ordinary biomechanical experiments, different internal fixation modes are mostly established with varying cadaver specimens, and only one fixation mode can be used for one specimen since implanting the metal internal fixation will damage the cadaver specimen; thus, there is individual variation between specimens [15]. The development of the FEA technique provides a solution. FEA was first used in the research on ankle diseases such as diabetic foot and flat foot deformity, and it is also widely applied in the design of artificial limbs, the residual artificial limb models, the plantar pressure measurement, and so on. FEA enables the analysis of the stress and 3D spatial displacement of bones and joints under different stress conditions [16]. Since the posterior subtalar joint is the majority of the whole subtalar joint, its fixation can realize the fixation of the whole subtalar joint, so subtalar fusion mainly involves the fixation of the posterior subtalar joint, and the anterior and medial subtalar joint needs no treatment [17]. Therefore, we only considered the stress distribution of the posterior subtalar joint in the 3D finite element analysis. During the experiment, we simplified the screw threads, confined the talus by exerting a pressure along the long axis of the screw, conducted the full-range simulation of double-screw fixation for the subtalar joint with the talar neck and posterior calcaneal tubercle as the range of screw insertion and by exerting a constant force on the calcaneus along the long axis of screw, and then analyzed the stress distribution changes after subtalar fusion.

The screw insertion with the most uniform stress distribution of the subtalar joint was found by 3D FEA. Combining the anatomic characteristics of the talus and calcaneus, the lateral screw was near the boundary of the talar neck and the medial screw was close to the talar surface of the ankle joint in the area of the talar neck. The former was located at the boundary of the posterior calcaneal tubercle and the latter was adjacent to the Achilles’ tendon terminal in the area of the posterior calcaneal tubercle. Therefore, inserting the lateral screw easily caused the split of the lateral talar and calcaneal bones, while inserting the medial screw easily injured the articular capsule, talar cartilage, and Achilles’ tendon terminal during the surgical procedures. It was seen that the screw insertion had disadvantages such as difficult localization and insertion and a big injury to adjacent tissues in the insertion area. In terms of the stress distribution uniformity, anti-rotary strength, and anti-inversion/eversion strength, the traditional longitudinally parallel screw insertion was inferior to the lateral-medial parallel screw insertion. When the screw was inserted toward the talus via the posterior calcaneal tubercle, the projection point of the screw was located on the talar surface of the ankle joint. Since the screw was inserted from the posterior calcaneal tubercle, the screw insertion position in the talar neck was not considered. The advantages were simple operation and easy localization, and the disadvantage was the possibility of injuring the talar surface if the selected screw length was unsuitable. The lateral-medial parallel screw insertion was worse than the screw insertion with the most uniform stress distribution in terms of the stress distribution uniformity, but comparable to the latter and superior to the traditional longitudinally parallel screw insertion in terms of the anti-rotary strength and anti-inversion/eversion strength. The advantages were that the talar neck and posterior calcaneal tubercle were divided in triplicate and the screws were inserted from the equal division points; the localization was simple, the insertion was convenient, and the insertion position was reliable and stable. The major movement of the subtalar joint was the inversion/eversion; the lateral-medial parallel screw insertion was vertical to its movement axis while the longitudinally parallel screw insertion was parallel to this axis. Thus, our independently designed lateral-medial parallel screw insertion demonstrated a stronger anti-rotary strength and anti-inversion/eversion strength in biomechanics than the traditional anteroposterior longitudinally parallel screw insertion. It was further confirmed by 3D finite element analysis.

Numerous clinical and biomechanical experiments have shown that the firm internal fixation is beneficial for improving the joint fusion rate, decreasing the infection rate, and promoting early functional training. Our follow-up results suggested that after operation, the AOFAS score was markedly increased, and the VAS scoring results indicated a significant pain relief in all patients. Muñoz [8] treated 37 patients by subtalar fusion with the Vira System, and the 1-year follow-up showed a fusion rate of 100% and mean fusion time of 14 weeks; in the AOFAS score, 9 cases were excellent (24.3%), 22 cases were good (59.4%), 5 cases were medium (13.5%), 1 case was poor (2%), and 1 case developed infection. Boffeli and Reinking [9] performed subtalar fusion with two screws by the posterior approach and suppedaneous approach in 31 patients; the fusion rate was 96%, the mean fusion time was 10.6 weeks, the pain was significantly relieved, and the postoperative function was satisfactory. One case developed metal reactive pain, 2 cases experienced wound pain, 1 case had foot tip numbness, and 1 case suffered lateral foot dorsum numbness. Albert and colleagues [10] treated 10 patients by subtalar fusion with two screws and the posterior approach; the mean follow-up time was 21.5 months, the fusion rate was up to 100%, the fusion time was 6.8 weeks (6–9 weeks), and the mean AOFAS score was increased to 78 after operation from 47 before operation; 2 cases experienced lateral foot dorsum numbness. Pollard and Schuberth [18] finished subtalar fusion with two screws and the posterior approach following bone grafting for calcaneal collapse fracture in 22 patients; the follow-up time was 27.3 months (12–63.9 months); 1 case had bony nonunion, and the fusion rate was 95.5%; 3 cases experienced wound numbness, and 7 cases suffered metal reactive pain. Compared with the above literature reports, the fusion rate in our study was up to 100%, and no complications such as screw loosening occurred, which is closely associated with the reliable and stable compression of double-screw insertion. Relative to traditional screw fixation approaches, the subtalar joint fixation technique used in this study demonstrated good postoperative recovery, few complications, and a high fusion rate. Such satisfactory clinical results may be correlated with the following factors: first, double-screw fixation can avoid the rotation of the subtalar joint in the 3D geometric space. One screw cannot prevent the subtalar fixation interface from rotation. de la Garza-Castro et al. [19] believed in their study of biomechanics that three screws were superior to two screws in the compression effect. The insertion of three screws was difficult because of mutual collision and increased the risk of wide soft tissue exposure, poor soft tissue healing, or infection, as well as the medical costs compared with that of two screws; thus, we selected double-screw fixation for subtalar fusion. Second, in our study, the talar neck and posterior calcaneal tubercle were divided in triplicate, so the position for screw insertion was easily localized, and the screw penetrated the compression interface with the maximum contact force during compression, thus providing a bigger torque and subsequently improving the anti-withdrawal strength or damage load [20].

## Conclusion

The lateral-medial parallel screw insertion not only demonstrates a good stress distribution profile of the subtalar joint but also has advantages such as easy localization and operation during surgery, as well as a high fusion rate and few complications after surgery. Therefore, it is a safe, accurate, and effective fixation mode which is worthy of being popularized clinically.

## Abbreviations

3D: Three-dimensional
AOFAS: American Orthopedic Foot & Ankle Society
SJ: Subtalar joint
VAS: Visual analogue scale

## References

[1] Hintermann B, Valderrabano V, Nigg B (2002). Influence of screw type on obtained contact area and contact force in a cadaveric subtalar arthrodesis model. Foot Ankle Int..

[2] Buckley R, Tough S, McCormack R, Pate G, Leighton R, Petrie D, Galpin R (2002). Operative compared with nonoperative treatment of displaced intra-articular calcaneal fractures: a prospective, randomized, controlled multicenter trial. J Bone Joint Surg Am.

[3] Tuijthof GJ, Beimers L, Kerkhoffs GM, Dankelman J, Dijk CN (2010). Overview of subtalar arthrodesis techniques: options, pitfalls and solutions. Foot Ankle Surg.

[4] Easley ME, Trnka HJ, Schon LC, Myerson MS (2000). Isolated subtalar arthrodesis. J Bone Joint Surg Am.

[5] Davies MB, Rosenfeld PF, Stavrou P, Saxby TS (2007). A comprehensive review of subtalar arthrodesis. Foot Ankle Int..

[6] Cheung JT, Zhang M, An KN (2004). Effects of plantar fascia stiffness on the biomechanical responses of the ankle-foot complex. Clin Biomech (Bristol, Avon).

[7] Coughlin MJ, Smith BW, Traughber P (2008). The evaluation of the healing rate of subtalar arthrodeses, part 2: the effect of low-intensity ultrasound stimulation. Foot Ankle Int..

[8] FL-O M. Design and development of an osteosynthesis system for minimally invasive reconstruction-arthrodesis of calcaneal intra-articular fractures. Rev Ortop Traumatol (Madr.). 2007;51(1):94–101.

[9] Boffeli TJ, Reinking RR (2012). A 2-screw fixation technique for subtalar joint fusion: a retrospective case series introducing a novel 2-screw fixation construct with operative pearls. J Foot Ankle Surg.

[10] Albert A, Deleu PA, Leemrijse T, Maldague P, Devos Bevernage B (2011). Posterior arthroscopic subtalar arthrodesis: ten cases at one-year follow-up. Orthop Traumatol Surg Res.

[11] Ma S, Jin D (2016). Isolated talonavicular arthrodesis. Foot Ankle Int.

[12] Behforootan S, Chatzistergos P, Naemi R (2017). Finite element modelling of the foot for clinical application: a systematic review. Med Eng Phys.

[13] Wong DW, Niu W, Wang Y (2016). Finite element analysis of foot and ankle impact injury: risk evaluation of calcaneus and talus fracture. PLoS One.

[14] Penix AR, Cook SD, Skinner HB, Weinstein AM, Haddad RJ Jr. Femoral head stresses following cortical bone grafting for aseptic necrosis. A finite element study. Clin Orthop Relat Res. 1983;(173):159-65. 6337757

[15] Lui TH, Chan LK (2010). Safety and efficacy of talonavicular arthroscopy in arthroscopic triple arthrodesis. A cadaveric study. Knee Surg Sports Traumatol Arthrosc.

[16] Perrier A, Bucki M, Luboz V, Vuillerme N, Payan Y. 3D musculoskeletal finite element analysis of the foot kinematics under muscle activation with and without ankle arthrodesis. Comput Methods Biomech Biomed Engin. 2015;14:1-2. 10.1080/10255842.2015.1069605 26273957

[17] Chapman JR, Harrington RM, Lee KM, Anderson PA, Tencer AF, Kowalski D (1996). Factors affecting the pullout strength of cancellous bone screws. J Biomech Eng.

[18] Pollard JD, Schuberth JM (2008). Posterior bone block distraction arthrodesis of the subtalar joint: a review of 22 cases. J Foot Ankle Surg..

[19] de la Garza-Castro S, Gonzalez-Rivera CE, Vilchez-Cavazos F (2017). Clinical, biomechanical and morphological assessment of anterior cruciate ligament Kevlar (R)-based artificial prosthesis in rabbit model. J Appl Biomater Funct Mater.

[20] Palmanovich E, Shabat S, Brin YS (2015). Anatomic reconstruction technique for a plantar calcaneonavicular (Spring) ligament tear. J Foot Ankle Surg.

